# Tracheal nebulization of AAV-CRE for lung conditional knockout model construction and whole lung clearing

**DOI:** 10.52601/bpr.2025.250005

**Published:** 2026-06-30

**Authors:** Lihong Ye, Xiyue Yan, Dacheng Yang, Shuang Wu, Wandong Chen, Wei Kevin Zhang

**Affiliations:** 1 Graduate School of Guangzhou Medical University, Guangzhou 511436, China; 2 Guangzhou National Laboratory, Guangzhou 510005, China

**Keywords:** Lung condition knockout, AAV, Cre-loxP mouse model, Tracheal nebulization, Whole lung clearing

## Abstract

The development of lung-specific conditional knockout animal models is pivotal in advancing lung disease research. This protocol describes the experimental procedures in detail, including the construction of loxP mice, endotracheal tube nebulization of adeno-associated virus (AAV) and whole lung clearing. Mouse lung conditional knockouts were successfully constructed. This meticulous approach allows for accurate control over the timing and location of the knockout, facilitating an in-depth investigation into the pathogenesis of lung diseases and the development of therapeutic strategies. These lung-specific conditional knockout models represent a powerful tool for the study of lung diseases, offering valuable insights and guidance for the advancement of treatment approaches and drug discovery in the future.

## INTRODUCTION

Transient receptor potential mucolipin 1 (TRPML1) is a cation channel encoded by the *Mcoln1* gene, located on the membranes of lysosomes and endosomes. It plays a critical role in intracellular calcium release and lysosomal function. Mutations in TRPML1 can cause a serious lysosomal storage disorder known as Mucolipidosis type IV (MLIV), marked by neurodegenerative alterations (Chen *et al.* 2017; Li *et al.* 2017). In Duchenne muscular dystrophy (DMD), TRPML1 agonists have been studied for their ability to enhance lysosomal function and the repair capacity of muscle cell membranes (Yu *et al.* 2020). Studies show that TRPML1 agonists can alleviate uranium-induced nephrotoxicity by promoting lysosomal excretion and biosynthesis (Zhong *et al.* 2023). Additionally, activation of TRPML1 can improve neuroprotection and functional recovery in amyotrophic lateral sclerosis (ALS) models by modulating autophagy functions and improving (Tedeschi *et al.* 2024). These findings indicate that TRPML1 holds significant therapeutic potential in regulating lysosomal function. Research concerning TRPML1 in relation to lung biology is still largely uninvestigated. Thus, exploring the regulatory mechanisms of TRPML1 in lung inflammation and immunity is crucial. This will aid in revealing its possible role in lung diseases and offer an essential theoretical framework and scientific basis for the future development and clinical use of TRPML1 agonists in pulmonary treatments.

The project employs principles of homologous recombination to modify the *Mcoln1* (Bach 2001; Misko *et al.* 1993) gene with flox (Moon *et al.* 2012; Sun *et al.* 2019) sequences in fertilized eggs. The procedure is outlined as follows: CRISPR-associated protein 9 (Cas9) mRNA and guide RNA (gRNA) are synthesized via *in vitro* transcription. A donor vector for homologous recombination is constructed using the In-Fusion cloning technique, incorporating a 3.0-kb 5' homologous arm, a 0.8-kb flox region, and a 3.0-kb 3' homologous arm. The Cas9 mRNA, gRNA, and donor vector are microinjected into fertilized eggs of C57BL/6J mice to produce the F0 generation. Positive F0 mice are identified through polymerase chain reaction (PCR) amplification and sequencing, and subsequently bred with C57BL/6J mice to generate positive F1 offspring. Breeding of F1 generation mice results in *Mcoln1*^*flox/flox*^ homozygous mice.

Adeno-associated virus (AAV) (El Andari and Grimm 2021; Santiago-Ortiz and Schaffer 2016) is widely used as a highly targeted and safe viral vector for gene delivery and expression, and is recognized as a promising gene therapy vector for the future. It is well known that the transduction efficiency of AAV is closely related to factors such as serotype and administration route. The use of AAV as a vector in gene therapy has progressed considerably, showing promising results in the treatment of genetic and neurological diseases. However, in clinical applications, AAV vectors still deal with challenges such as immune responses and the potential risks of gene integration. Hence, the development of preclinical models that more closely mimic human physiology is critical for AAV gene therapy studies. Humanized mouse models are essential in the study of AAV gene therapy. These models involve transplanting human cells or tissues into immunodeficient mice, and provide a physiological environment akin to humans for analyzing the transduction efficiency and immune response of AAV vectors (Ye and Chen 2022).

Recent research efforts have been directed at improving the specificity and efficiency of this technology, for example, AAV9.452sub.LUNG1 has shown significantly enhanced transgene expression in lung tissue following systemic delivery in mice, emphasizing the promise of customized AAV vectors for lung-targeted gene therapy (Goertsen *et al.* 2022). Efforts in capsid engineering are directed towards refining the delivery of AAV vectors to the lungs, with intra-tracheal administration of AAV6 vectors showing persistent transduction in the lungs of mice without genomic integration. This approach tackles safety concerns about insertional mutagenesis. It allows for high and sustained gene expression, making it a promising strategy for therapies aimed at the lungs (Colon-Cortes *et al.* 2020).

The pulmonary nebulizer is a drug nebulizer designed for pulmonary administration in small animals, consisting of a high-pressure-resistant syringe and a spray needle. During administration, the spray needle is inserted orally into the trachea, and the syringe is manually operated to deliver the drug. The pulmonary nebulizer can effectively achieve quantitative drug delivery into the trachea without wastage.

### Development of the protocol

This plan primarily encompasses the construction of loxP (locus of X-over P1) mice, nebulization of the AAV virus, and whole lung clearing. The outlined methodology offers a systematic approach for constructing lung-specific conditional knockout (CKO) models targeting specific cell types. The process involves determining the appropriate AAV serotype, promoter, and viral dosage. Subsequently, determine the mechanical parameters of tracheal nebulization, followed by an investigation into the distribution patterns within the pulmonary system. Additionally, perform whole lung clearing on successfully modeled lungs to evaluate nebulization efficiency. In summary, this detailed protocol facilitates the construction of lung-specific conditional knockout animal models.

### Application and advantages of the protocol

This is a systematic plan for lung-specific conditional knockout, which allows for the rapid and efficient construction of animal models with specific cell types knocked out in the lungs. Conditional knockout mouse models are typically obtained through at least two generations of breeding transgenic animals, which is time-consuming and costly. In contrast, the cost of AAV-CRE (Cyclization Recombination Enzyme) injection is much lower, and the expression begins approximately seven days post-injection. By ensuring tissue-specific infection through localized injection and utilizing a CRE gene with a specific promoter, enhanced tissue and cell-specific gene recombination is achievable. Through the modification and optimization of serotypes and promoters, alongside improvements in viral packaging efficiency, AAV demonstrates superior knockout efficiency. The benefits of AAV-CRE can be summarized as follows: shorter cycle, lower cost, strong spatial specificity, time-specific control, and high recombination efficiency. The pulmonary nebulizer is made of stainless steel, allowing for cleaning, high-temperature sterilization, and high-pressure disinfection. It can deliver a precise amount of medication into the lungs, working by direct insertion into the trachea or bronchi to avoid drug loss and waste. Compared to traditional methods of drug administration, such as dripping, it provides a more uniform distribution of medication.

Tracheal intubation combined with nebulized delivery of AAV gene vectors holds significant importance. Nebulization technology enables the conversion of AAV vectors into fine aerosol particles, facilitating non-invasive and highly efficient gene delivery. This approach is particularly suitable for the treatment of respiratory diseases, as it allows direct delivery of gene vectors to targeted regions of the lungs, thereby reducing systemic exposure and potential side effects (Qi *et al.* 2009).

Large tissue clearing technology is becoming more recognized for its applications and advantages in lung research. Firstly, the use of this technology not only enhances research precision but also provides new perspectives for the diagnosis and treatment of lung diseases. Tissue clearing-based methods enable cellular-level imaging of entire organs without the need for sectioning (Rong *et al.* 2025). For instance, recent studies have demonstrated that clearing techniques can achieve three-dimensional imaging of the entire pulmonary vascular system, thereby improving our understanding of tumor angiogenesis and distribution (Gong *et al.* 2024). Secondly, tissue clearing technology plays a significant role in evaluating lung drug delivery systems. By combining tissue clearing with advanced microscopic imaging techniques, researchers can observe the three-dimensional microstructure of entire lung tissues with unprecedented resolution. This not only helps uncover the mechanisms of lung diseases but also provides a foundation for developing new therapeutic approaches (Zhu *et al.* 2013). In summary, the application of large tissue clearing technology in lung research offers scientists a clearer and more comprehensive perspective. The continuous advancement of this technology will further drive progress in lung disease research.

### Limitations of the protocol

1　This protocol is suitable for AAV delivery in mouse pulmonary systems, with the success rate of model establishment being highly dependent on the proficiency of the experimenter in executing nebulization procedures.

2　The administration of AAV-CRE may lead to heterogeneous distribution. Therefore, in practical applications, the incorporation of fluorescent proteins should be considered to enhance the accuracy and efficacy of sample preparation.

3　Subsequent assessments of pulmonary function in the murine lung knockout model, utilizing techniques such as micro-computed tomography (micro-CT) and pulmonary function testing instruments, may pose a risk of inducing mechanical damage to the lungs.

### Overview of the protocol

First, construct the *Mcoln1*^*flox/flox*^ mouse model. Next, establish the knockout animal model through intubation aerosol delivery of AAV-CRE. Subsequently, evaluate the nebulization efficiency using tissue clearing and frozen sectioning. Simultaneously, assess the gene knockout efficiency using immunofluorescence staining. Overall, this protocol is designed to facilitate the rapid and effective construction of a controllable knockout animal model, with advantages in terms of time and space.

## SUMMARIZED PROCEDURE

### *Mcoln1*^*flox*^ mouse preparation

1　Transcribe Cas9 mRNA and gRNA *in vitro*.

2　Construct a donor vector containing homologous arms and flox region through In-Fusion cloning.

3　Microinject Cas9 mRNA, gRNA, and donor vector into fertilized eggs of C57BL/6J mice to generate F0 mice.

4　Heterozygous F0 mice were identified by performing PCR amplification and sequencing.

5　Hybrid F0 mice were mated to breed F1 mice.

6　Mice identified as positive by PCR (flox/flox) and wild type (wt) can be used for the next step of the experiment.

### Mouse tail genotyping

1　Design of PCR amplification primers. Primers should be designed to flank the loxP site, with one primer located upstream at the 5' end and the other downstream at the 3' end, taking into account the integration of loxP sites within the target gene.

2　Preparation of fresh lysate. Combine lysate with proteinase K in a 50:1 ratio.

3　Sample preparation. Place the excised mouse tail in an 8-tube strip, add 100 µL of fresh lysate to each sample, and centrifuge at 2000 r/min for 30 s.

4　PCR instrument programming. Set the program to incubate at 55°C for 20 min, followed by denaturation at 95°C for 5 min.

5　Sample processing. Briefly centrifuge the samples, after which the supernatant can be directly utilized for PCR reactions.

6　Thaw 2× Taq mix on ice, and prepare the reaction system on ice as follows: ddH_2_O 11.5 μL, 2× Taq mix 12.5 μL, Lysate 1 μL, Primer F (100 μmol/L) 0.1 μL, and Primer R (100 μmol/L) 0.1 μL.

7　Set up PCR reaction program. (1) 94°C for 5 min; (2) 94°C for 30 s; (3) 55°C for 30 s; (4) 72°C for 30 s/kb; (5) 72°C for 10 min. Repeated Steps (2)−(4) for 35 cycles.

8　Agarose gel preparation. Prepare a 1.5% agarose gel using 1× Tris Acetate-EDTA (TAE) buffer as the electrophoresis buffer.

9　Electrophoresis. Conduct horizontal electrophoresis on the amplified products.

10 Screening. Screen for *flox/flox* and *wt/wt* genotypes in mice for subsequent experiments.

### Endotracheal tube atomization

1　AAV Preparation: Thaw the AAV on ice, gently vortex, briefly centrifuge, and maintain on ice. The injection dose for each mouse was set at 1 × 10^11^ vg, supplemented to 50 µL with saline. The virus was aspirated using a nebulizer and reserved for later use.

2　Mice were injected intraperitoneally with tribromoethanol at 200 µL per 10 g body weight.

3　Mice were fixed on the operating table with heads up and limbs down, and the operating table was adjusted to be perpendicular to the ground.

4　Use a laryngoscope to open the throat and observe the opening and closing of the vocal cords. Insert the nebulizer needle into the middle of the vocal cords. Remove the laryngoscope, adjust the nebulizer needle to be parallel to the trachea, and push the nebulizer needle with appropriate force.

5　Continue to care for the mice.

### Whole lung sampling

1　Mice sacrificed by orbital exsanguination.

2　Place the mouse on the experimental operating table.

3　Cut the chest cavity of the mouse with disinfected scissors and forceps to expose the lungs.

4　Dissect the heart, esophagus, and adipose tissue, and carefully remove the lungs to avoid damaging the surrounding tissues.

5　Lungs were placed on glass slides, and the expression abundance and location of fluorescent tags carried by AAV were observed under an upright microscope.

6　Cut tissue as needed.

7　Fixation with 4% Perfluoroalkoxy (PFA), stored at 4°C for one day.

### Lung clearing

1　Remove PFA and wash lungs three times for 4 h each with phosphate buffer saline (PBS) on a shaker at 100 r/min.

2　Degrease with Solution 1 from the kit, changing every three days for three times. The volume of Solution 1 should be five times that of the tissue. 100 r/min, 37°C.

3　Rinse with PBS, changing every three hours for threetimes. 100 r/min, 37°C.

4　Further decolorization and degreasing with actual and medium Solution 2, replacing every three days for three times. The volume of Solution 2 should be five times that of the tissue. 100 r/min, 37°C.

5　Rinse with PBS, changing every three hours for three times. 100 r/min, 25°C.

6　Perform refractive index matching with Solution 3 from the kit. The volume of Solution 3 should be three times that of the tissue. Replace once a day for 2 d at room temperature.

7　Store samples in Solution 3, protected from light, at room temperature.

### Preparation steps of frozen section

1　Lung tissue sampling and fixation. Take out the target tissue block and place it in 4% paraformaldehyde. Fix it at 4°C for 2 h.

2　Washing. Wash the tissue once with 1× PBS for 5 min.

3　Dehydration. Place the tissue in a 30% sucrose solution for sedimentation and dehydration, usually for 24 h.

4　Embedding. Put the dehydrated tissue block into the optimal cutting temperature compound (OCT) for embedding.

5　Freezing. Rapidly freeze the embedded tissue block in liquid nitrogen for 3−5 min, then transfer it to −80°C for storage.

6　Sectioning. Fix the frozen tissue block on the microtome, set the section thickness (usually about 10 μm), and perform sectioning.

7　Mounting. Attach the cut tissue sections to a glass slide. You can use a cold stage or warm water to help the sections adhere to the slide.

8　Storage. Frozen sections need to be stored at −80°C and can be preserved for several months.

### Preparation steps of paraffin section

1　Lung tissue sampling and fixation. Take out the target tissue block and place it in a fixative solution (commonly 4% paraformaldehyde or 10% formalin). The amount of the fixative solution should be more than five times the volume of the tissue. The fixation time is usually 12 to 24 h.

2　Trimming and rinsing. Trim the tissue block according to the requirements, put it in an embedding cassette, and rinse it with running tap water for 30 min to wash away the residual fixative solution.

3　Dehydration. Place the tissue successively in gradient ethanol solutions (75%, 80%, 90%, 95%, 100%) for 30 min each time.

4　Clearing. Put the dehydrated tissue in xylene for clearing treatment, 20 min each time, for a total of two times.

5　Impregnation with paraffin. Place the cleared tissue in molten paraffin for 90 min each time, for a total of three times.

6　Embedding. Put the paraffin-impregnated tissue block into molten paraffin and let it cool and solidify to form a paraffin block.

7　Sectioning.

(A) Preparation. Place the paraffin block on an ice stage for 10 to 30 min to increase its hardness for easier sectioning.

(B) Sectioning. Fix the paraffin block on the microtome, set the section thickness (usually 5 μm), and perform sectioning.

(C) Spreading. Put the cut paraffin sections into warm water at 45 to 50°C. The paraffin sections will naturally spread out due to the surface tension.

(D) Lifting. Use a glass slide to lift up the spread paraffin sections, mark the relevant information, and place them on a staining rack.

(E) Baking (optional). Put the glass slide in a 60°C constant-temperature oven for half an hour to make the paraffin sections adhere firmly to the slide.

### Immunofluorescence (frozen section)

#### Preparation of experimental materials and reagents

1　Sections. The thickness of frozen sections is generally 8−10 μm.

2　Fixative. 4% paraformaldehyde.

3　Washing solution. PBS buffer solution.

4　Antigen retrieval solution. EDTA antigen retrieval buffer solution (pH 8.0).

5　Blocking solution. 1% BSA (bovine serum albumin).

6　Antibodies.

(A) Primary antibody. Select an appropriate immunofluorescence-grade primary antibody according to the target protein.

(B) Secondary antibody. A fluorescently labeled secondary antibody that matches the primary antibody.

7　Other reagents.

(A) Tissue autofluorescence quenching agent.

(B) DAPI (used for nuclear staining).

(C) Antifade mounting medium.

#### Experimental steps

1　Section treatment.

(A) Take out the frozen sections from the refrigerator and let them recover to room temperature for 20−30 min.

(B) Fix the sections in 4% paraformaldehyde for 30 min.

(C) Wash the sections with PBS buffer solution three times, 5 min each time.

2　Antigen retrieval (optional).

(A) Immerse the sections in the EDTA antigen retrieval buffer solution.

(B) Put them in a microwave oven for antigen retrieval. Heat on medium power for 8 min until boiling, stop heating and keep warm for 8 min, then heat on medium-low power for 7 min.

(C) After natural cooling, wash with PBS buffer solution three times, 5 min each time.

3　Blocking and permeabilization.

(A) Draw a circle around the tissue on the section with a histochemical pen, drop Solution A of the tissue autofluorescence quenching agent, and incubate at room temperature for 30 min.

(B) Wash with pure water for 5 min.

(C) Drop the 1% BSA blocking solution and incubate at room temperature for 30 min.

4　Incubation with primary antibody.

(A) Gently shake off the blocking solution and drop the diluted primary antibody.

(B) Place the sections flat in a humid chamber and incubate at 4°C overnight.

5　Washing and incubation with secondary antibody.

(A) Immerse the sections in PBS buffer solution and wash three times, 5 min each time.

(B) Drop the fluorescently labeled secondary antibody and incubate at room temperature for 50 min in the dark.

(C) Wash with PBS buffer solution three times, 5 min each time.

6　Counterstaining (optional).

(A) Drop the ready-to-use DAPI staining solution and incubate at room temperature for 10 min in the dark.

(B) Wash with PBS buffer solution three times, 5 min each time.

7　Mounting and storage.

(A) Drop the antifade mounting medium on the section and mount it with a coverslip.

(B) Store in the dark at 4°C or −20°C.

8　The staining was photographed using an upright fluorescence microscope.

**[CAUTION!]** (1) Antibody selection. Ensure that immunofluorescence-grade antibodies are used. (2) Operation in the dark. Fluorescent dyes are easily bleached by light, so the operation should be carried out in the dark. (3) Section moisturization. Avoid the sections from drying out, and the incubation should be carried out in a humid chamber. (4) Condition optimization. Optimize the incubation time and temperature according to the antibody and tissue type.

### HE staining (paraffin section)

1　Dewaxing and hydration.

(A) Xylene I: Soak for 10 min.

(B) Xylene II: Soak for 10 min.

(C) 100% Ethanol: Soak for 5 min.

(D) 95% Ethanol: Soak for 5 min.

(E) 80% Ethanol: Soak for 5 min.

(F) 70% Ethanol: Soak for 5 min.

(G) Distilled Water: Soak for 5 min.

2　Hematoxylin staining.

(A) Hematoxylin staining solution. Stain for 3−8 min.

(B) Rinse with tap water for 5 min.

(C) Differentiation with 1% hydrochloric acid ethanol for a few seconds.

(D) Rinse with tap water for 5 min.

(E) Blue with 0.6% ammonia water for 5−10 s.

(F) Rinse with tap water for 5 min.

3　Eosin staining.

(A) 0.5%−1% eosin staining solution. Stain for 1−3 min.

(B) Rinse with tap water for 30 s.

4　Dehydration and clearing.

(A) 70% ethanol. Soak for 30 s.

(B) 80% ethanol. Soak for 1 min.

(C) 90% ethanol. Soak for 1 min.

(D) Absolute ethanol (I). Soak for 3 min.

(E) Absolute ethanol (II). Soak for 3 min.

(F) Xylene (I). Soak for 3 min.

(G) Xylene (II). Soak for 3 min.

5　Mounting. Wipe off the excess xylene around the section, drop an appropriate amount of neutral balsam, and cover with a coverslip for fixation.

**[CAUTION!]** (1) Thorough dewaxing. Incomplete dewaxing will affect the staining effect. (2) Differentiation time of hematoxylin. The differentiation time needs to be adjusted according to the tissue type and staining effect. (3) Staining time of eosin. Excessive staining time will make the cytoplasm color too dark. (4) Operation environment. The whole process should be carried out in a well-ventilated environment.

### Anticipated results

**Figure 1:**
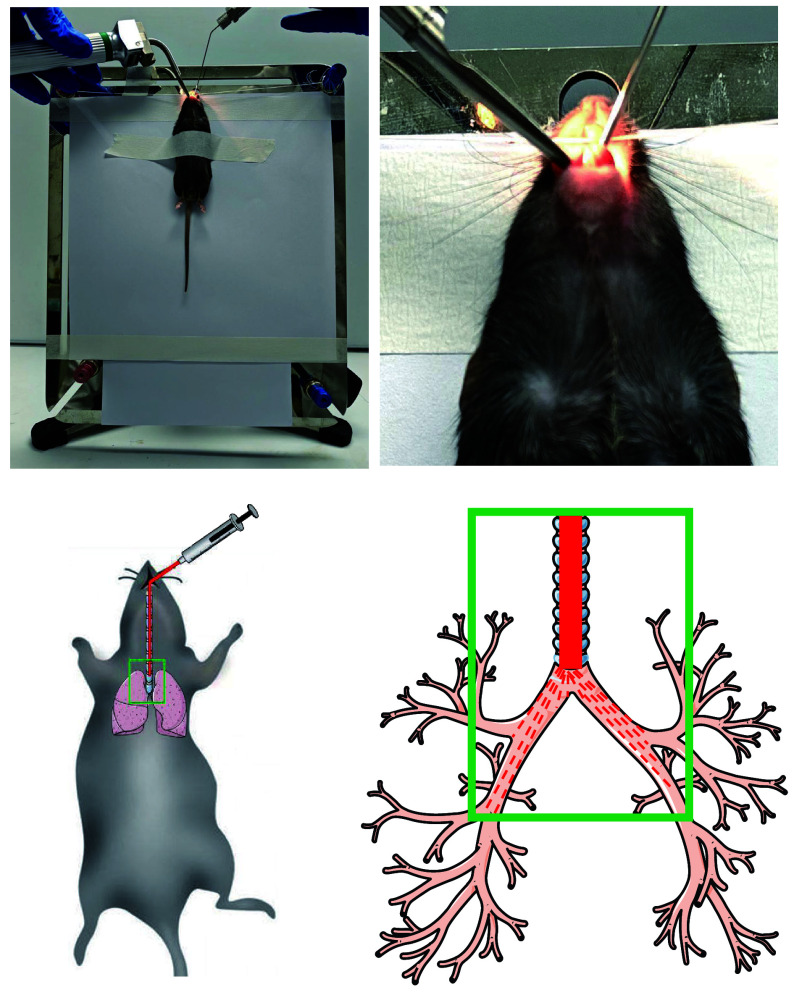
Schematic diagram of tracheal intubation nebulization. One hand uses a laryngoscope to open the trachea, and expose the vocal cords, while the other hand inserts the nebulization needle into the trachea. Adjust the nebulization needle to the appropriate position and push the needle to complete the nebulization process

In accordance with the established protocol, we initially procured mice that underwent consistent lung nebulization of AAV via tracheal intubation nebulization. Figure 1 illustrates the operational process. The mice were positioned vertically on the operating table, with their incisors suspended on the line, limbs fixed, and the airway opened with a laryngoscope. Subsequently, the nebulization needle was inserted into the trachea, with the insertion position located 0.5 cm above the position of the heartbeats, to complete the nebulization.

**Figure 2:**
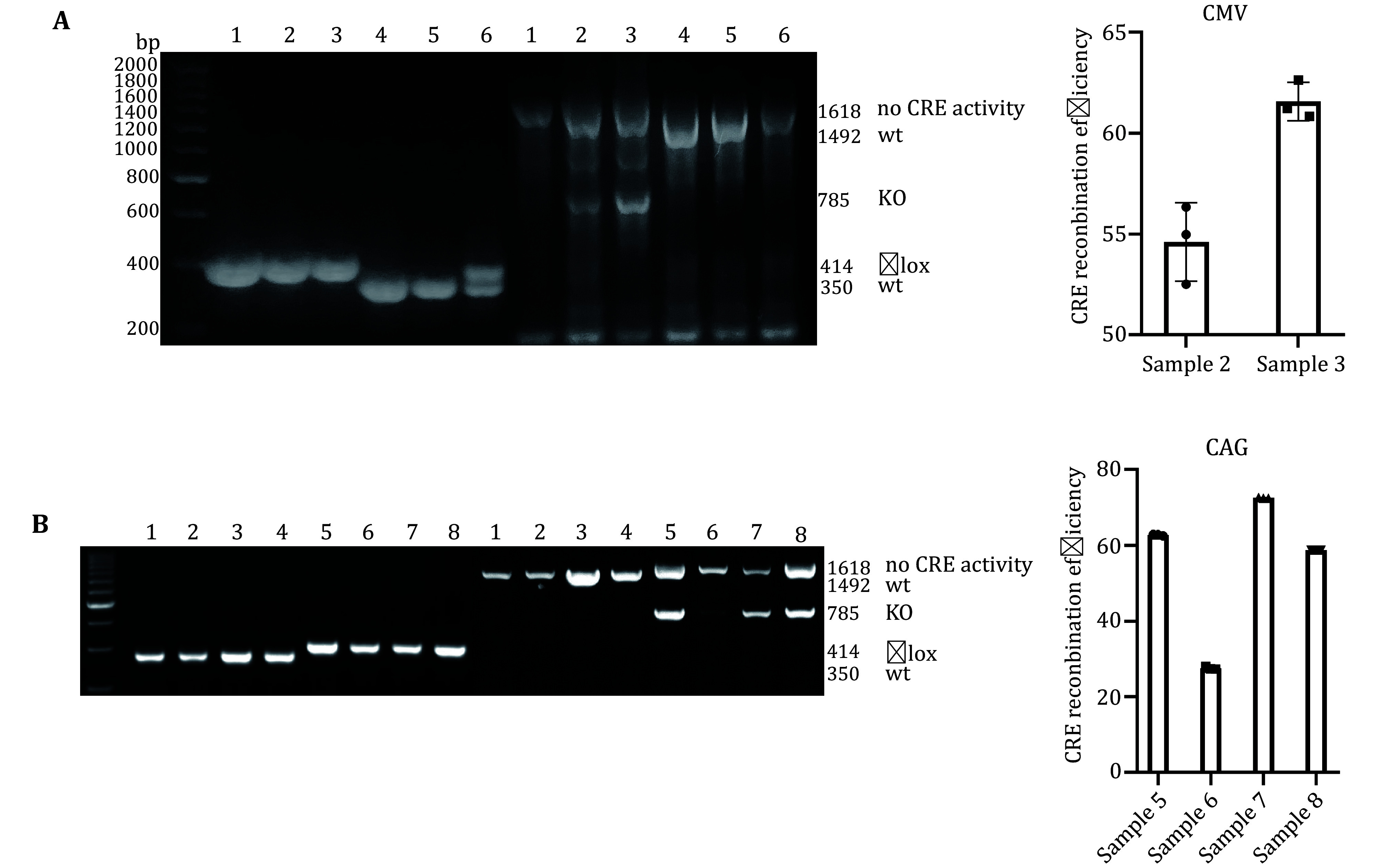
Genotyping of lung tissue. Different genotypes were determined by PCR detection of bands of varying lengths: samples with CRE activity exhibited a band at 785 bp, samples without CRE activity exhibited a band at 1618 bp, and the wild type exhibited a band at 1492 bp. The fluorescence intensity of the bands was quantified using ImageJ. **A** Genotyping results of lung tissue from C57 mice treated with nebulized AAV (AAV 2/6.2-CMV-EGFP-P2A-CRE-WPRE-pA). **B** Genotyping results of lung tissue from C57 mice treated with nebulized AAV (AAV 2/6.2-CAG-ZsGreen-P2A-CRE-WPRE-pA)

**Figure 3:**
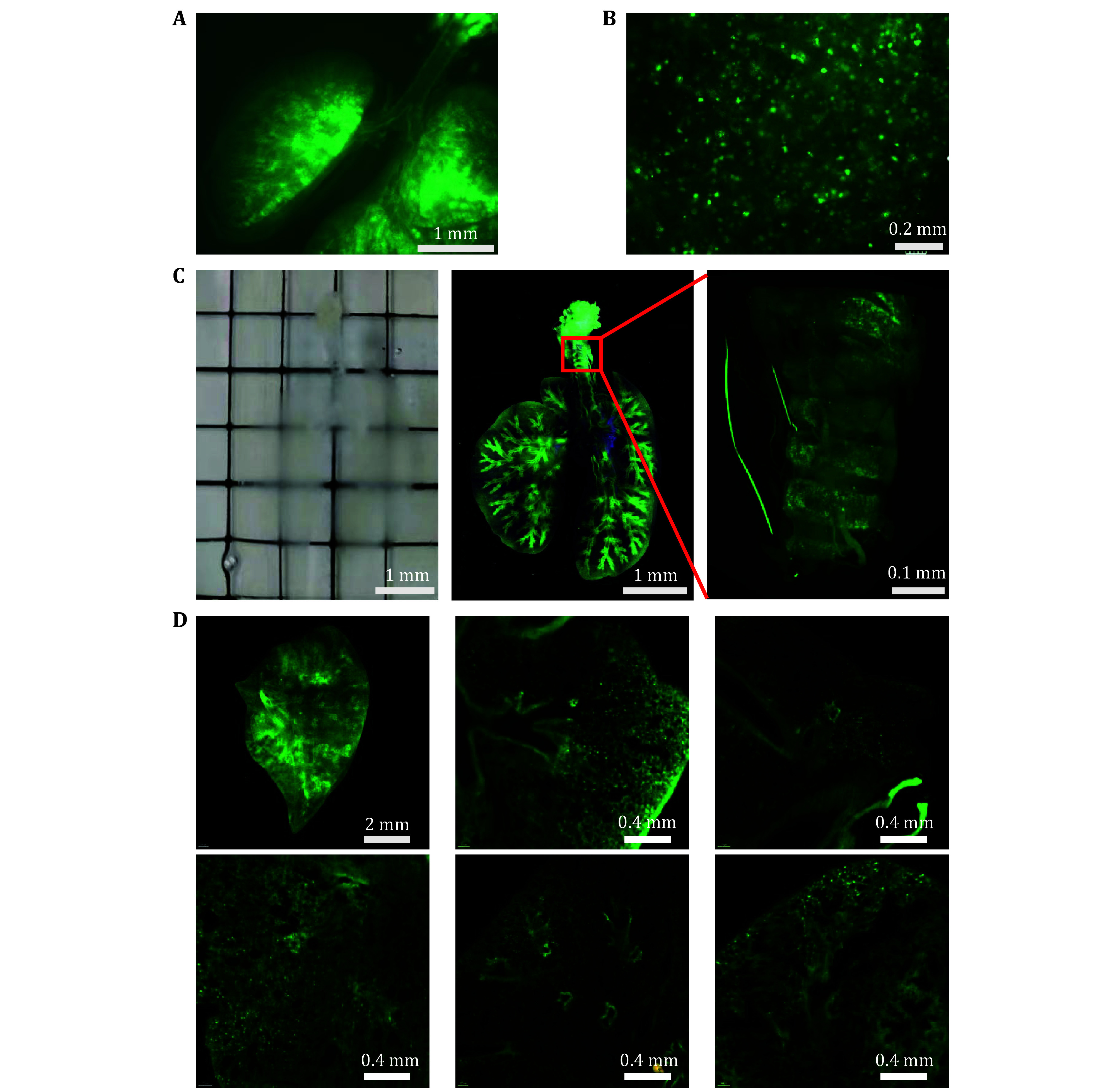
**A** The image observed under a stereomicroscope after delivering AAV(AAV 2/6.2-CMV-EGFP-P2A-CRE-WPRE-pA) labeled with EGFP(enhanced green fluorescent protein) to the lungs via nebulization, followed by whole-lung clearing processing. **B** A fluorescent image of lung tissue from C57 mice. Flox mice were aerosolized with AAV carrying the CAG promoter and ZsGreen green fluorescent label. Each mouse was given a virus dose of 1E11. After two weeks of expression, the fresh tissue was imaged under a brightfield microscope. **C** Three-dimensional reconstruction image of whole lung clearing. C57 mice were treated with tracheal intubation aerosolization. The AAV serotype is 6.2, with the CMV promoter carrying the EGFP tag. After two weeks of expression, the entire lung was fixed and cleared. **D** Single-page three-dimensional reconstruction image and sectional display of the lung. Selective knockout of AT1 was performed using the *SPB* promoter and carrying the ZsGreen label. As shown in the figure, successfully infected cells can be clearly observed in the distal lung and alveoli

**Figure 4:**
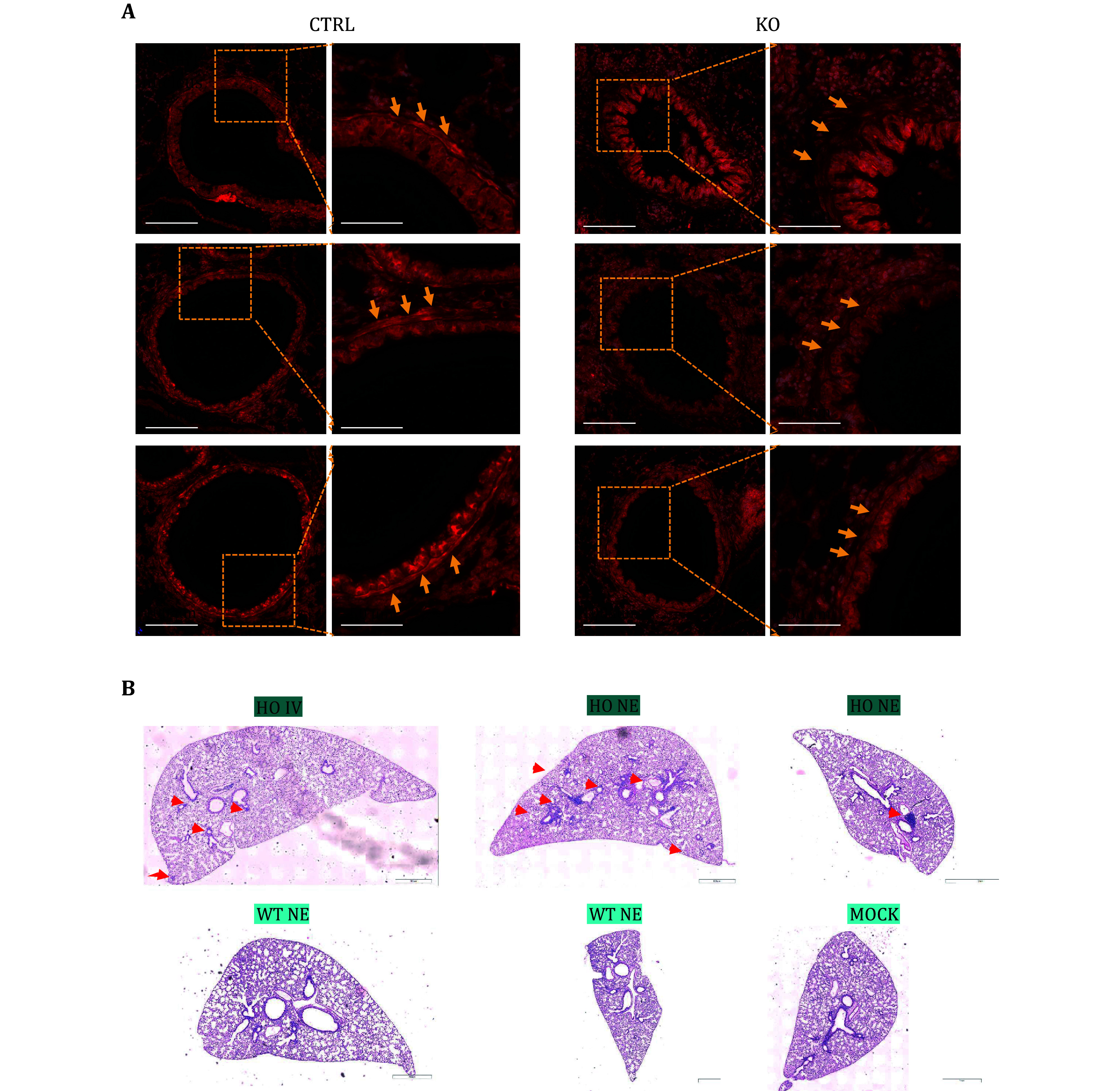
**A** Immunofluorescence staining results. Nebulized AAV carrying CRE protein reduced the expression of Mcoln1 in lung tissue. **B** HE staining results. Conditional knockout of Mcoln1 showed significant inflammatory infiltration around the bronchi

After two weeks of expression, the AAV vector achieved the desired knockout model. As shown in Fig. 2, lung tissue was taken for genomic PCR identification, and the PCR product band for CKO mice was enriched at 785 bp, demonstrating a successful knockout effect of AAV. Figure 3A demonstrates the imaging results of the entire lung after tissue clearing, revealing the alveolar structure. Figure 3B displays the fresh lung tissue under an optical microscope. The CAG promoter is a broad-spectrum promoter that can express in most cells. This experiment carried a ZsGreen fluorescent label, which has a fluorescence intensity four times that of EGFP. As shown in the figures, most tissues expressed green fluorescence, and the fluorescence was evenly distributed. This indicates that our nebulized AAV model achieved spatial control and temporal efficiency in lung conditional knockout, making it an efficient experimental method. Figure 3C shows the effect of whole lung clarification. Clarification is a decolorization and degreasing process, followed by refractive index matching to achieve overall transparency. In this experiment, a broad-spectrum cytomegalovirus promoter (CMV) was selected, showing a high AAV infection effect in the trachea and bronchi. After the whole lung clarification, the distribution of the virus throughout the lung can be observed. As shown in the figures, tracheal intubation nebulization achieved the goal of AAV infection throughout the lung. The local magnified image of the trachea demonstrates the highly efficient infection capability of AAV. Figure 3D illustrates a type I alveolar cell (AT1) cell-specific knockout model. Surfactant protein B (SPB) is a protein specifically expressed in AT1 cells. Using the SPB promoter, the specific knockout of the target protein in AT1 cells can be achieved. As shown in the figures, specific expression of the fluorescent protein ZsGreen can be observed in AT1 cells in the distal airways and alveoli, demonstrating the successful achievement of gene knockout in specific cell populations. Further histological results are shown in Fig. 4. Tissues are prepared into paraffin sections for immunofluorescence staining or hematoxylin and eosin (HE) staining. The results showed that the protein level of TRPML1 in bronchial smooth muscle cells was upregulated, and significant inflammatory infiltration was observed around the trachea.

## MATERIALS

### Reagents

• Pathogen-free C57BL/6 male mice were available from the Shanghai Model Organisms Center

• PBS (Cytiva, SH30256.01)

• Tribromoethanol (Nanjing AIBI bio-Technology Co., Ltd, 2407A)

• PFA (Properich, 120452)

• 75% alcohol (Macklin, C16178982)

• Genotyping kit (Vazyme, Cat. No. PD101-01)

• Clearing kit (Nuohai Life Science, Cat. No. NH210701)

• Saline (NMPA, No. H43020455)

### Primer sequences

• *Mcoln1* flox F: CCTACGTCCTTCCCACTTGT

• *Mcoln1* flox R: AGCTTGATCCTCTGAACCCA

• *Mcoln1* cre work F: TACTCTCCCAGACGTCCCTG

• *Mcoln1* cre work R: AAGGTGGGTACAGGAGTGGT

### Equipment

• Upright fluorescence microscope (Guangzhou Huiteng Science & Technology Co., BA410)

• Pulmonary nebulizer (TOW-INT TECH, DP-M)

• Animal operating table (TOW-INT TECH, ST)

• 1 mL Syringes (CFDA, 0.45×16RWLB)

• Instrument for Polymerase Chain Reaction PCR (Trident960)

### Abbreviations

**Table:** 

AAV	Adeno-associated virus
ALS	Amyotrophic lateral sclerosis
AT1	Type I alveolar cell),
Cas9	CRISPR-associated protein 9
CKO	Conditional knockout
CMV	Cytomegalovirus promoter
CRE	Cyclization recombination enzyme
DMD	Duchenne muscular dystrophy
gRNA	Guide RNA
HE	Hematoxylin and eosin
loxP	Locus of X-over P1
micro-CT	Micro-computed tomography
MLIV	Mucolipidosis type IV),
OCT	Optimal cutting temperature
PBS	Phosphate buffer saline
PCR	Polymerase chain reaction
PFA	Perfluoroalkoxy
SPB	SurfactantproteinB
TRPML1	Transient receptor potential mucolipin 1
